# Testicular Vasculitis: A Sonographic and Pathologic Diagnosis

**DOI:** 10.1155/2017/8923621

**Published:** 2017-01-26

**Authors:** Anuj Dixit, Cameron Hague, Simon Bicknell

**Affiliations:** ^1^Discipline of Radiology, Memorial University of Newfoundland, St. John's, NL, Canada; ^2^Department of Radiology, University of British Columbia, Vancouver, BC, Canada

## Abstract

Very little has been published about single-organ vasculitis of the testicle in the radiological literature. Consequently, it is a diagnosis that is unfamiliar to most radiologists. This case report describes the sonographic, pathologic, and laboratory findings of testicular vasculitis and reviews the available literature with regard to this subject.

## 1. Introduction

Systemic vasculitides can often involve the testes; however, isolated vasculitis of the testes is uncommon [1]. When a clinical history suggesting an underlying vasculitis is not present in the setting of testicular pain, the diagnosis is a difficult one. Although the imaging features are often nonspecific, in the right clinical setting, it is a diagnosis that the radiologist may be able to suggest.

## 2. Case Presentation

An 84-year-old male presented to the emergency department with testicular pain worsening over a 24-hour period. The patient was otherwise healthy with no significant medical concerns and no other symptoms. A testicular ultrasound was arranged on an urgent basis with the differential diagnosis consisting of epididymoorchitis versus torsion.

**Figure 1:**
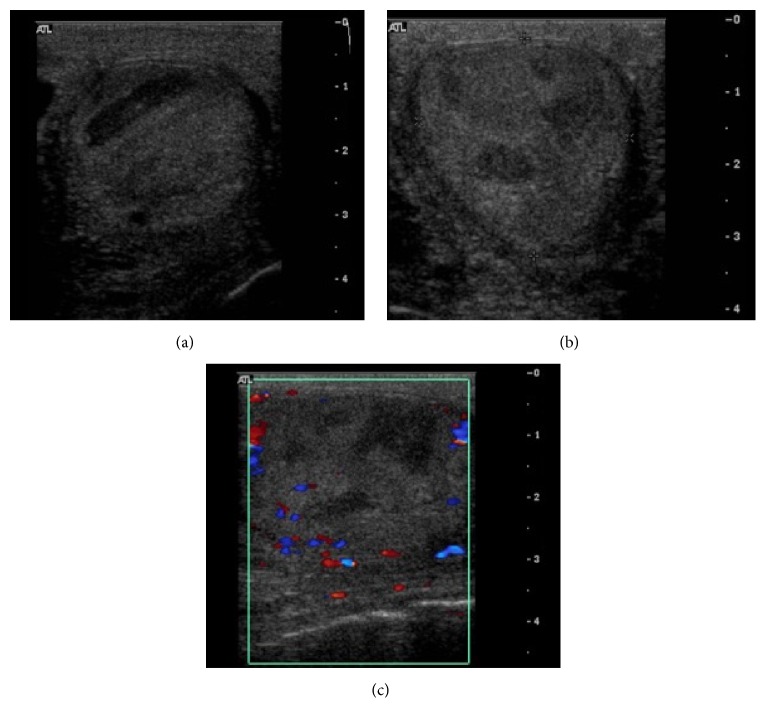
Multiple axial and longitudinal sonographic images (a–c) of the left testicle with Doppler color demonstrating a heterogeneous appearance, with multiple hypoechoic mass-like areas, and lack of Doppler flow within the majority of the testis. The right testis (image not provided) had a similar appearance.

Sonographic evaluation revealed a heterogeneous appearance of both testicles with diminished parenchymal Doppler flow (Figure 1). The preliminary diagnosis was testicular infarction secondary to torsion or a neoplastic process such as lymphoma. Surgical excision of the left testicle was arranged.

**Figure 2:**
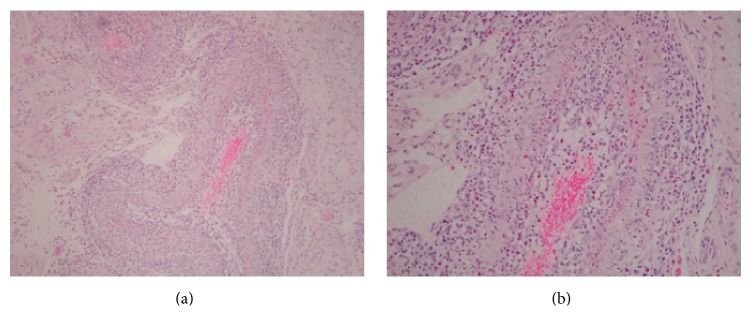
Histological section (H&E stain) of the left testicle viewed at medium (a) and high (b) power demonstrating a proliferation of neutrophils as well as T and B lymphocytes surrounding one of the intratesticular vessels consistent with vasculitis. No granulomas are present.

**Figure 3:**
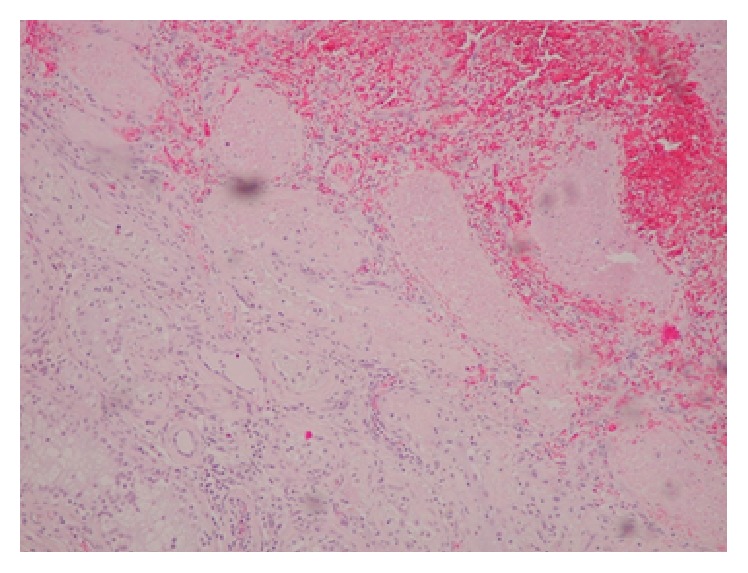
Histological section (H&E stain) of the left testicle viewed at medium power demonstrating hemorrhagic infarction of seminiferous tubules secondary to underlying vasculitis.

The specimen was submitted for pathologic evaluation, which revealed a medium vessel vasculitis with associated hemorrhagic infarction of much of the testicular parenchyma (Figures 2 and 3). Vasculitic inflammatory change was also visualized in the regions of the epididymis and spermatic cord. No granulomas were seen and no evidence of lymphomatous or leukemic infiltrates were identified.

Given the pathological findings, additional blood work to assess an underlying vasculitis was obtained. The antinuclear antibody (ANA) screen was negative. The anti-neutrophil cytoplasmic antibody (ANCA) indirect immunofluorescence (IIF) was positive with a perinuclear pattern (p-ANCA). Anti-proteinase 3 (PR3-ANCA) and anti-myeloperoxidase (MPO-ANCA) antibody testing by ELISA (INOVA Diagnostics Inc.) were both negative. Protein electrophoresis revealed decreased albumin and beta 1 (LDL and transferrin) and beta 2 (C3) globulins. Midstream urinalysis was unremarkable. CRP was elevated at 72 mg/L (reference range < 10 mg/L). The patient was hepatitis B and hepatitis C negative. Liver function tests were normal. HIV status was not determined, but the patient had no known risk factors. Given the medium vessel involvement demonstrated on pathological assessment, as well as the blood work, a diagnosis of nongranulomatous testicular vasculitis was made. Clinical workup for the presence of systemic vasculitis was negative and inflammatory markers returned to normal values following orchiectomy and medical management.

Unfortunately, the patient was lost to follow-up following discharge which we acknowledge is a limitation of this case report.

## 3. Discussion

Various forms of vasculitis can involve the testes. While PAN is the most common form to affect the testicle, granulomatosis with polyangiitis (GPA), Henoch-Schonlein purpura, giant cell arteritis, and vasculitis associated with some autoimmune connective tissue disorders such as Systemic Lupus Erythematosus (SLE) can also involve the testes [1, 2]. A recent study found the majority of cases of testicular vasculitis (TV) involve the testicular parenchyma while a lesser proportion of cases involved the epididymis (44.6%) and spermatic cord (30.6%) [3].

PAN was first described by Kussmaul and Maier in 1866 and commonly affects multiple organs in a patient such as the skin, kidneys, gastrointestinal tract, and peripheral and central nervous systems [4]. PAN is a medium-sized vessel vasculitis predominantly affecting males in their 4th to 6th decade. It is associated with a positive hepatitis B surface antigen serology in 10–50% of cases. PAN is also associated with positive HIV serology. Testicular involvement by PAN was first reported in the early 1900s [5]. Since then, isolated PAN has been observed in the gallbladder, uterus, skin, lungs, breast, and kidneys [6].

In 2012, the Chapel Hill consensus for nomenclature of vasculitides added “single-organ vasculitis” as a new category to differentiate PAN which is reserved for the primary systemic form of this medium-sized vessel vasculitis [7]. As such, isolated organ involvement which pathologically shows identical to PAN was to be categorized as single-organ vasculitis (in our case, testicular vasculitis).

Laboratory results, in addition to pathological findings, are crucial in establishing a specific vasculitis as the causative factor for the testicular findings seen on ultrasound. ANA positivity is suggestive of a diagnosis of SLE or other connective tissue diseases. ANCA positivity is helpful in identifying certain small vessel vasculitides. A cytoplasmic pattern (c-ANCA) by IIF and PR-3 positivity by ELISA are suggestive of GPA. A p-ANCA by IIF and MPO positivity by ELISA are suggestive of microscopic polyangiitis (MPA). Our patient had a positive p-ANCA by IIF and a negative PR3 and MPO by ELISA, supporting a diagnosis of PAN-type rather than GPA or MPA. It should be noted that PAN is not classically associated with ANCA [8]. As such, PAN can be diagnosed in patients with c-ANCA, p-ANCA, or nonspecific nuclear ANCA results on immunofluorescence pattern testing. Again, as there was no systemic evidence of vasculitis, our case was classified as testicular vasculitis (TV) or medium-sized vessels vasculitis of the testicle.

There is no consensus regarding the treatment of TV, although it is postulated that the excision of the affected organ is curative [3, 9, 10]. This is important to contrast to a systemic vasculitis with testicular involvement (most commonly PAN) where the treatment involves pharmacologic therapy. The complete absence of systemic symptoms and normal laboratory results suggest no need for further invasive diagnostic procedures, such as renal, skin, or muscle biopsies [4].

As mentioned previously, the sonographic diagnosis of SOV affecting the testicle is a difficult one. Little has been published about the sonographic appearances of testicular vasculitis in the radiological literature [11], likely contributing to the fact that it is a diagnosis that the radiologist may be unlikely to consider if no history of an underlying vasculitis is provided. Furthermore, the majority of published cases report isolated testicular vasculitis occurring in young patients [4, 12]. Our case is the first describing such findings in a male greater than 80 years of age.

Testicular vasculitis is a great mimic [1, 11, 13]. It can appear sonographically normal or heterogeneous with variable Doppler flow or may present with multiple mass-like intratesticular lesions [1, 13]. Given its variable appearance, it may be prudent to keep vasculitis on the list of differential possibilities when such nonspecific testicular findings are seen. Subacute testicular torsion and neoplastic etiologies including primary testicular malignancy, metastatic disease, and lymphoma should be considered in addition to testicular vasculitis, particularly if sonographic evaluation demonstrates mass-like intratesticular findings and altered Doppler flow.

It should be noted that a limitation of this study was the lack of follow-up of the patient. It is true that, in any medium-sized vessel vasculitis, P-ANCA positivity at IIF (even in the absence of MPO specificity by ELISA) should alert the clinician for the possible systemic extent (even subclinical), which can also evolve to a generalized disease over time.

## 4. Conclusion

The presented case provides an important teaching point with regard to the differential diagnosis of nonspecific testicular sonographic findings in patients with testicular pain. Due to a relative lack of literature addressing testicular vasculitis, it may be a diagnosis that is overlooked. In the right clinical setting, even given the lack of specific ultrasound findings, testicular vasculitis is an entity that should be considered.
